# Multifunctional Polyhedral Oligomeric Silsesquioxane (POSS) Based Hybrid Porous Materials for CO_2_ Uptake and Iodine Adsorption

**DOI:** 10.3390/polym13020221

**Published:** 2021-01-10

**Authors:** Mohamed Gamal Mohamed, Mei-Yin Tsai, Chih-Feng Wang, Chih-Feng Huang, Martin Danko, Lizong Dai, Tao Chen, Shiao-Wei Kuo

**Affiliations:** 1 Department of Materials and Optoelectronic Science, Center of Crystal Research, National Sun Yat-Sen University, Kaohsiung 80424, Taiwan; mgamal.eldin34@gmail.com (M.G.M.); m073100011@student.nsysu.edu.tw (M.-Y.T.); 2 Chemistry Department, Faculty of Science, Assiut University, Assiut 71516, Egypt; 3 Advanced Membrane Materials Research Center, Graduate Institute of Applied Science and Technology, National Taiwan University of Science and Technology, Taipei 10607, Taiwan; cfwang@mail.ntust.edu.tw; 4 Department of Chemical Engineering, i-Center for Advanced Science and Technology (iCAST), National Chung Hsing University, Taichung 40227, Taiwan; huangcf@dragon.nchu.edu.tw; 5 Department of Synthesis and Characterization of Polymers, Polymer Institute, Slovak Academy of Sciences, Dúbravská cesta 9, 84541 Bratislava, Slovakia; martin.danko@savba.sk; 6 Fujian Provincial Key Laboratory of Fire Retardant Materials, College of Materials, Xiamen University, Xiamen 361005, China; lzdai@xmu.edu.cn; 7 Ningbo Institute of Material Technology and Engineering, Chinese Academy of Science, Ningbo 315201, China; tao.chen@nimte.ac.cn; 8 Department of Medicinal and Applied Chemistry, Kaohsiung Medical University, Kaohsiung 807, Taiwan

**Keywords:** octavinylsilsesquioxane, Friedel–Crafts reaction, porous polymers, CO_2_ capture, iodine adsoprtion

## Abstract

In this study, two different types of hybrid porous organic polymers (POPs), polyhedral oligomeric silsesquioxane tetraphenylpyrazine (POSS-TPP) and tetraphenylethene (POSS-TPE), were successfully synthesized through the Friedel−Crafts polymerization of tetraphenylpyrazine (TPP) and tetraphenylethene (TPE), respectively, with octavinylsilsesquioxane (OVS) as node building blocks, in the presence of anhydrous FeCl_3_ as a catalyst and 1,2-dichloroethane at 60 °C. Based on N_2_ adsorption and thermogravimetric analyses, the resulting hybrid porous materials displayed high surface areas (270 m^2^/g for POSS-TPP and 741 m^2^/g for POSS-TPE) and outstanding thermal stabilities. Furthermore, as-prepared POSS-TPP exhibited a high carbon dioxide capacity (1.63 mmol/g at 298 K and 2.88 mmol/g at 273 K) with an excellent high adsorption capacity for iodine, reaching up to 363 mg/g, compared with the POSS-TPE (309 mg/g).

## 1. Introduction

The preparation of porous organic polymers (POPs) has become a hot topic in the academic and industry fields due to their interesting features, such as high physicochemical stability, porous character, low density, facile preparation, low regeneration energy, and good thermal and chemical stabilities [1,2,3,4,5,6,7,8,9,10,11,12,13,14,15]. Porous organic polymers have been applied in different applications such as light harvesting, chemical sensors, catalysis, iodine uptake, H_2_ production from water, water treatment, optoelectronic devices, carbon dioxide reduction, nanofiltration, enantioseparation, energy storage, gas separation, and adsorption [14,15,16,17,18,19,20,21,22,23,24,25,26,27,28,29]. The synthesis of POPs was successfully achieved using various synthetic methods, including Friedel–Crafts arylation, Schiff base reactions, Suzuki reactions, Yamamoto reactions, Heck reactions, and Sonogashira reactions [30,31,32,33,34,35,36,37,38,39,40,41]. In addition, POPs can be classified into different kinds of materials, such as covalent organic frameworks (COFs), conjugated microporous polymers (CMPs), hypercrosslinked polymers (HCPs), covalent triazine-based frameworks (CTFs), metal–organic frameworks (MOFs), and polymers of intrinsic microporosity (PIMs) [37,38,39,40,41,42,43,44,45,46,47,48,49,50,51,52].

Polyhedral oligomeric silsesquioxanes (POSSs) are a kind of nanostructured compound with a diameter in the range of 1–3 nm, an empirical formula (*R*_8_Si_8_O_12_), a well-defined structure, and a controlled porosity [53,54,55,56,57,58,59,60,61]. Recently, using an inorganic POSS as a building block for preparation, hybrid porous materials have attracted much attention because of their excellent thermal and mechanical proprieties, water retardance, and chemical stability [62,63,64,65,66]. As reported, hydrosilylation, Heck, Friedel–Crafts, Yamamoto, and Sonogashira reactions could be used for the preparation of POSS porous materials [67,68]. For example, Liu et al. successfully prepared a porous polymer (Fc-HPP) through the Friedel–Crafts reaction of ferrocene (Fc) with OVS. These materials displayed a high surface area (1015 m^2^/g) and excellent performance for removing heavy metals and dyes [66]. Liu et al. also prepared a series of porous polymers derived from OVS with various ethene derivates through the heck coupling reaction [67]. The same group also used a Friedel–Crafts reaction to prepare multifunctional hybrid porous materials from OVS and tetraphenylene for metal ion detection and dye adsoprtion. They revealed that these materials possess dual pore structures with a high surface area (up to 1910 m^2^/g) [65].

In this work, we designed, synthesized, and applied in CO_2_ and I_2_ the capture of two different types of hybrid porous organic polymers (POSS-TPP and POSS-TPE) derived from the Friedel−Crafts polymerization of tetraphenylpyrazine (TPP) and tetraphenylethene (TPE), respectively, with octavinylsilsesquioxane (OVS) as node building blocks in the presence of anhydrous FeCl_3_ as a catalyst and 1,2-dichloroethane at 60 °C. Various instruments, such as Fourier-transform infrared spectroscopy (FTIR), solid state ^13^C cross-polarization/magic-angle spinning (CP/MAS), nuclear magnetic resonance spectroscopy (NMR), Brunauer–Emmet–Teller theory (BET), XRD, TEM, and SEM were used to examine the chemical structures, morphology, crystallinity, and porous properties for POSS-TTP and POSS-TPE, respectively. The results revealed that the as-prepared POSS-TPP exhibited a high carbon dioxide capacity (1.63 mmol/g at 298 K and 2.88 mmol/g at 273 K) with an excellent and high adsorption capacity for iodine, reaching to up to 363 mg/g.

## 2. Materials and Methods

### 2.1. Materials

Octavinylsilsesquioxane (OVS, 98%), benzophenone (99%), anhydrous ferric chloride (FeCl_3,_ 99.99%), 1,2-dichloroethane (DCE, 99.8%), anhydrous magnesium sulfate (MgSO_4_, 99.5%), ammonium acetate (CH_3_COONH_4_, 98%), acetic acid (CH_3_COOH, 99%), potassium carbonate (K_2_CO_3_, 99.995%), and benzoin (98%) were purchased from Alfa Aesar (Heysham, United Kingdom). Tetrahydrofuran (THF), acetone, methanol (CH_3_OH), and dichloromethane (DCM) were ordered from Aldrich (Taipei, Taiwan).

### 2.2. Synthesis of Tetraphenylpyrazine (TPP)

TPP was synthesized as reported in the previous study with a minor modification [69]. Benzoin (2.12 g, 10 mmol), acetic anhydride (1.45 mL, 15 mmol), ammonium acetate (2.32 g, 30 mmol), and 50 mL of acetic acid were added to a 50 mL round-bottom flask. After refluxing for 3.5 h at 90 °C, the mixture was cooled down to room temperature and then filtered. The crude products were purified with recrystallization in acetic acid to afford TPP as a white powder (yield: 34%) (^1^H NMR (500 MHz, δ, ppm, CDCl_3_, Figure S1): 7.65 (m, 8H), 7.33 (m, 12H); ^13^C NMR (125 MHz, δ, ppm, CDCl_3_, Figure S2): 148.5, 138.5, 129.9, 128.7, 128.3).

### 2.3. Synthesis of Tetraphenyethene (TPE)

TPE was prepared according to our previous study [7,13]. Benzophenone (3.00 g, 16.20 mmol), zinc (3.91 g, 59.4 mmol), and dry THF (100 mL) were stirred and kept at 0 °C for 30 min. Then, TiCl_4_ (3.29 mL, 29.4 mmol) was added slowly to the reaction mixture at 0 °C, and the reaction solution was refluxed at 80 °C for 18 h. After that, a 10% aqueous solution of K_2_CO_3_ was added dropwise to the reaction mixture at room temperature. The reaction mixture was extracted with ethylacetate (EA), dried over MgSO_4_ and filtered, and the THF solution was removed to yield TPE as a white crystalline solid (yield: 97%) (^1^H-NMR (500 MHz, δ, ppm, CDCl_3_, Figure S3): 7.05–7.15 (m, 20H); ^13^C-NMR (125 MHz, δ, ppm, CDCl_3_, Figure S4): 140.7, 141.0, 131.3, 127.7, 126.4).

### 2.4. Synthesis of POSS-TPP

A solution of OVS (0.63 g, 1 mmol), tetraphenylpyrazine (0.32 g, 0.8 mmol), and FeCl_3_ (0.9 g, 6.7 mmol) in dry 1,2-dichloroethane (20 mL) was prepared. The mixture was stirred at room temperature for 0.5 h and then heated under reflux at 60 °C for 24 h. After cooling at room temperature, the solid was filtered and washed three times with THF, methanol, chloroform, and acetone to remove the unreacted monomer and FeCl_3_. Then, it was dried under a vacuum at 80 °C to get a red-brown solid (0.55 g, 87%).

### 2.5. Synthesis of POSS-TPE

A solution of OVS (0.63 g, 1 mmol), TPE (0.28 g, 0.8 mmol), and FeCl_3_ (0.9 g, 6.7 mmol) in 1,2-dry dichloroethane (20 mL) was stirred at room temperature for 0.5 h and then heated under reflux at 60 °C for 24 h. After cooling at room temperature, the solid was filtered and washed three times with THF, methanol, chloroform, and acetone to remove the unreacted monomer and FeCl_3_. It was then dried under a vacuum at 80 °C to get a red-brown solid (0.59 g, 93%).

### 2.6. Uptake of Iodine

The procedure of iodine uptake by both porous materials (POSS-TPP and POSS-TPE) was carried out as follows: POSS-TPP or POSS-TPE (5 mg) was suspended in 10 mL of an I_2_ solution in hexane. The solution mixture was stirred at different times, and then the UV-Vis measurement was done. The calibration curve of the I_2_ solution was recorded by using various iodine concentrations.

## 3. Results

### 3.1. Synthesis of POSS-TPP and POSS-TPE

**Scheme 1:**
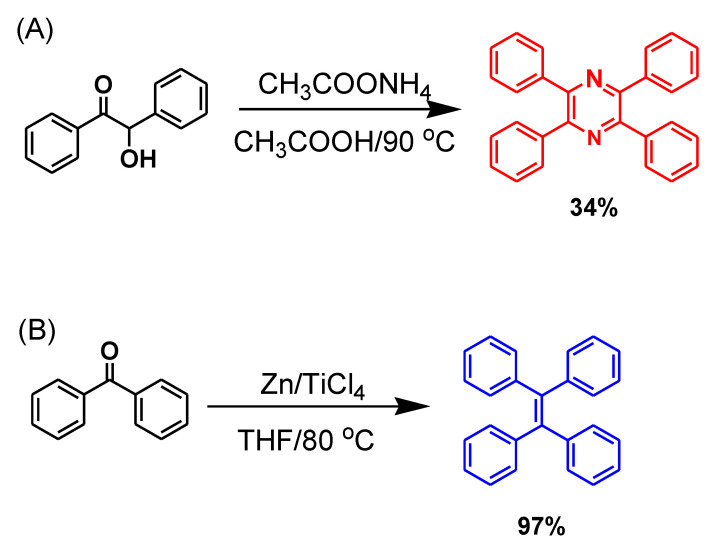
Synthesis of (**A**) tetraphenylpyrazine (TPP) and (**B**) tetraphenylethene (TPE).

**Scheme 2:**
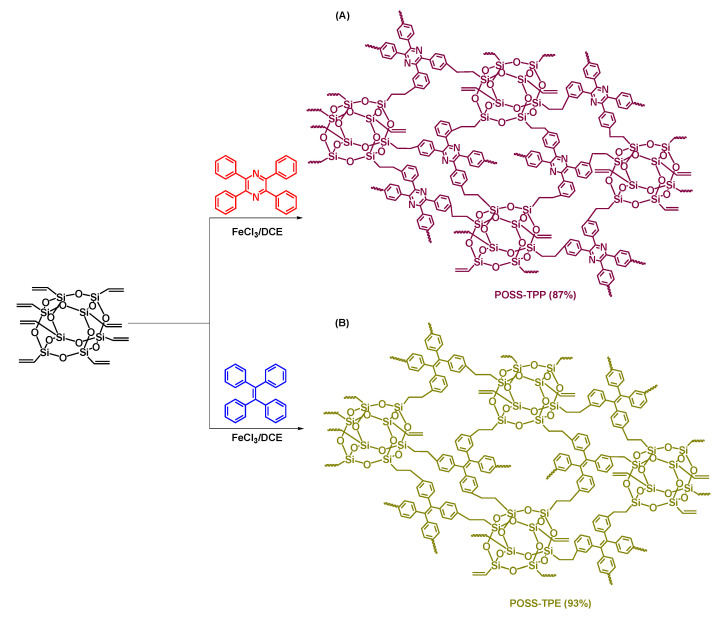
Synthesis of (**A**) polyhedral oligomeric silsesquioxane tetraphenylpyrazine (POSS-TPP) and (**B**) polyhedral oligomeric silsesquioxane tetraphenylethene (POSS-TPE).

**Figure 1:**
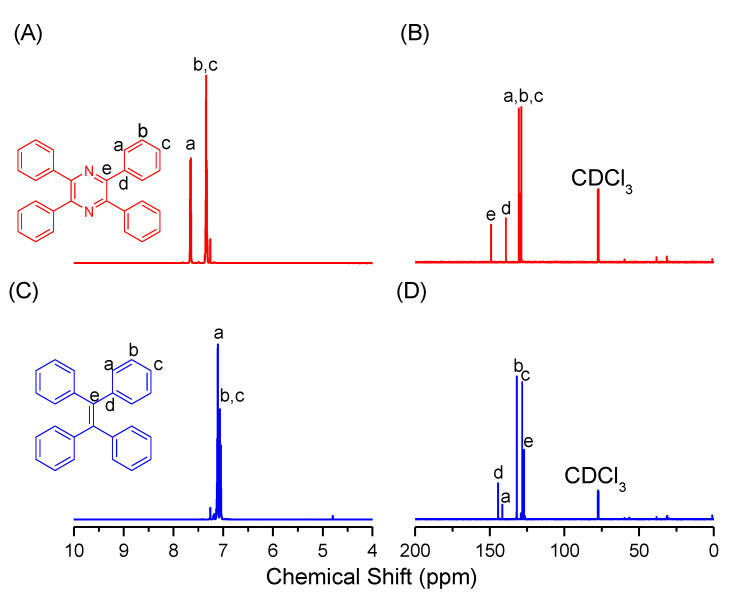
^1^H-NMR spectra of (**A**) POSS-TPP and (**B**) POSS-TPE, and the ^13^C-NMR spectra of (**C**) POSS-TPP and (**D**) POSS-TPE.

The preparation route of the TPP and TPE monomers is shown in Scheme 1. The TPP monomer was successfully synthesized by the reaction of benzoin with ammonium acetate (CH_3_COONH_4_) in the presence of acetic acid at 90 °C. The TPE monomer was prepared by the reaction of benzophenone with zinc in dry THF as a solvent and the presence of TiCl_4_ at 80 °C under an N_2_ atmosphere. While the schematic method for the preparation of two different hybrid microporous polymers (POSS-TPP and POSS-TPE) was based on OVS, TPP and TPE are shown in Scheme 2. Firstly, POSS-TPP was prepared by a Friedel–Crafts reaction of OVS with TPP in the presence of anhydrous FeCl_3_ as a catalyst and 1,2-dichloroethane as solvent at 60 °C for 24 h in an N_2_ atmosphere (Scheme 2A), while POSS-TPE was synthesized by a Friedel–Crafts reaction of OVS with TPE in the presence of anhydrous FeCl_3_ as a catalyst and 1,2-dichloroethane as a solvent at 60 °C for 24 h under an N_2_ atmosphere (Scheme 2B). The resulting materials were successively washed several times with dimethylformamide (DMF), dimethylacetamide (DMAc), THF, DCM, and MeOH to remove FeCl_3_ and unreacted OVS, as well as other monomers. In addition, the POSS-TPP and POSS-TPE were not soluble in acetone, THF, DMF, DCM, or MeOH. Figure 1 shows the ^1^H-NMR spectra of TPP and TPE with CDCl_3_ as the solvent. The proton signals were located at 7.64 ppm and 7.35 ppm for the aromatic rings in the TPP (Figure 1A). The ^1^H-NMR spectrum (Figure 1B) of TPE showed a characteristic proton signal centered at 8.04–7.04 ppm for the aromatic proton signals. Furthermore, we used ^13^C-NMR measurements to confirm the chemical structures of the TPP and TPE, as displayed in Figure 1. As shown in Figure 1C,D, the signals of the aromatic carbon nuclei in the TPP and TPE moieties appeared in the range of 149.64–129.29 ppm for the TPP and 141.06–126.40 ppm for the TPE.

**Figure 2:**
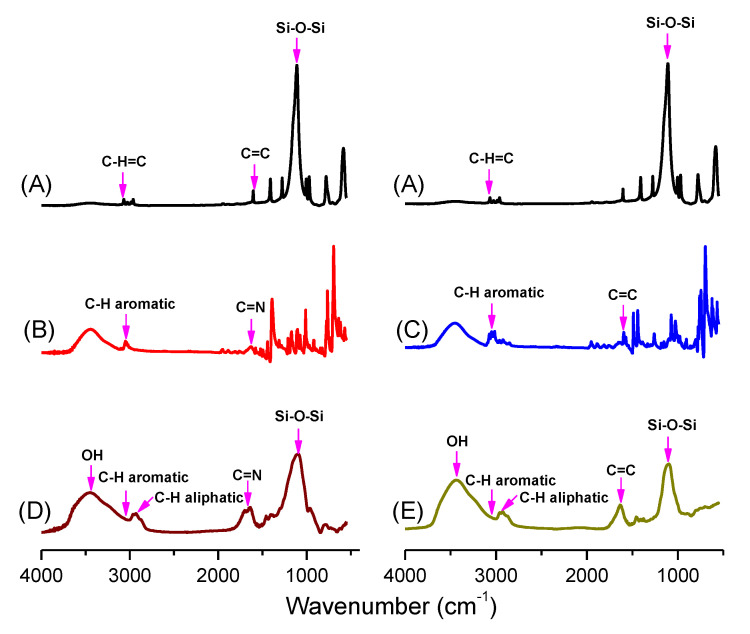
Fourier-transform infrared spectroscopy (FTIR) spectra of (**A**) octavinylsilsequioxane (OVS), (**B**) TPP, (**C**) TPE, (**D**) POSS-TPP, and (**E**) POSS-TPE.

In addition, FTIR analyses were used to investigate the functional groups in OVS, TPP, TPE, POSS-TPP, and POSS-TPE, respectively (Figure 2). The absorption bands of Si–O–Si, C=C–H, and C=C in the OVS (Figure 2A) were located at 1107 cm^−1^, 1600 cm^−1^, and 3066 cm^−1^, respectively. The FTIR spectra of TPP, TPE, POSS-TPP, and POSS-TPE displayed a characteristic absorption band in the range of 3048–3052 cm^−1^ for the stretching C–H aromatic groups and at 1599 cm^−1^ for the C=C bonds (Figure 2B–E). In addition, the absorption signals appeared at 3450 cm^−1^, 2950 cm^−1^, and 1457 cm^−1^ in the FTIR spectra of POSS-TPP and POSS-TPE (Figure 2D,E), representing adsorbed water by our porous materials and the presence of CH_2_-CH_2_ linkage in both the POSS-TPP and POSS-TPE polymer frameworks.

**Figure 3:**
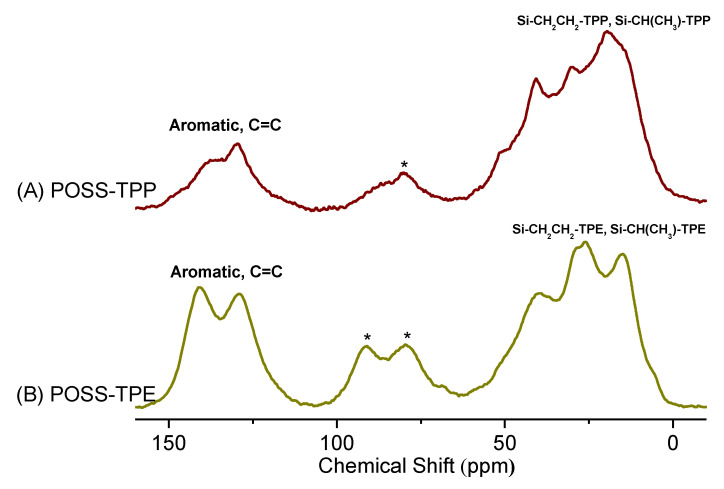
Solid-state ^13^C CP/MAS NMR spectra of the states of (**A**) POSS-TPP and (**B**) POSS-TPE, where * is the side band of solid state nuclear magnetic resonance spectroscopy (NMR).

The chemical structures of POSS-TPP and POSS-TPE were confirmed through the solid-state ^13^C CP/MAS analyses (Figure 3). The solid-state ^13^C CP/MAS profiles (Figure 3) of POSS-TPP and POSS-TPE features carbon nuclei signals in the range 138.11–129.77 ppm and 41.08–20.80 pm for POSS-TPP (Figure 3A) which corresponds to aromatic ring in POSS-TPP, unreacted vinyl carbons, and Si–CH_2_–CH_2_-TPP or Si–CH(CH_3_)-TPP units, respectively. While the signals appearing in the range 140.94–129.17 and 39.96–15.17 pm for POSS-TPE (Figure 3B) arising from TPE units, unreacted vinyl carbons and Si–CH_2_–CH_2_-TPE or Si–CH(CH_3_)-TPE units, respectively. 

**Figure 4:**
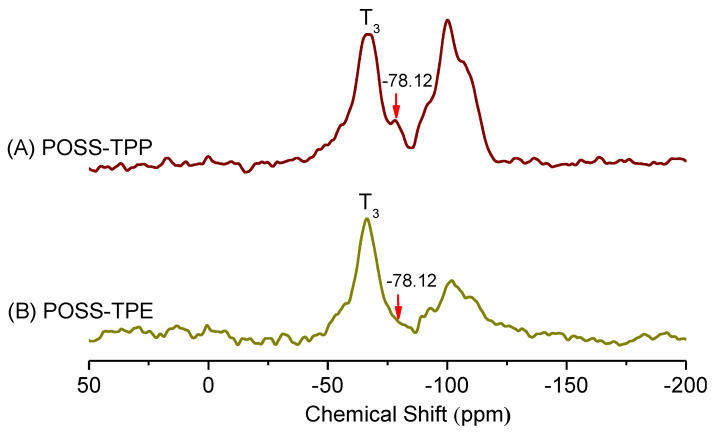
^29^Si spectra of (**A**) POSS-TPP and (**B**) POSS-TPE.

The solid state ^29^Si MAS NMR measurement was done to confirm the presence of POSS moiety in both POSS-TPP and POSS-TPE, as presented in Figure 4. In the displayed solid state ^29^Si MAS NMR spectra of POSS-TPP and POSS-TPE, two major signals at −66.95 ppm and −78.66 ppm were attributed to the silicon atom of CSi(OSi)_3_ (T_3_) from the SiCH_2_ CH_2_TPP, Si¬CH(CH_3_) TPP units and the unreacted SiCH=CH_2_ group, respectively, and for POSS-TPP and the silicon atom of CSi(OSi)_3_ (T_3_) from the SiCH_2_CH_2_TPE, SiCH(CH_3_) TPE units and unreacted SiCH=CH_2_ group, respectively. In addition, the disappearance of signals arising from the T_1_ or T_2_ units (T_n_ = CSi (OSi)_n_(OH)_3−n_) indicated both the remaining structure and the lack of cleavage of the cage structure in the POSS-TPP and POSS-TPE hybrid polymers.

### 3.2. Thermal Stability and Porosity Properties of POSS-TPP and POSS-TPE

**Figure 5:**
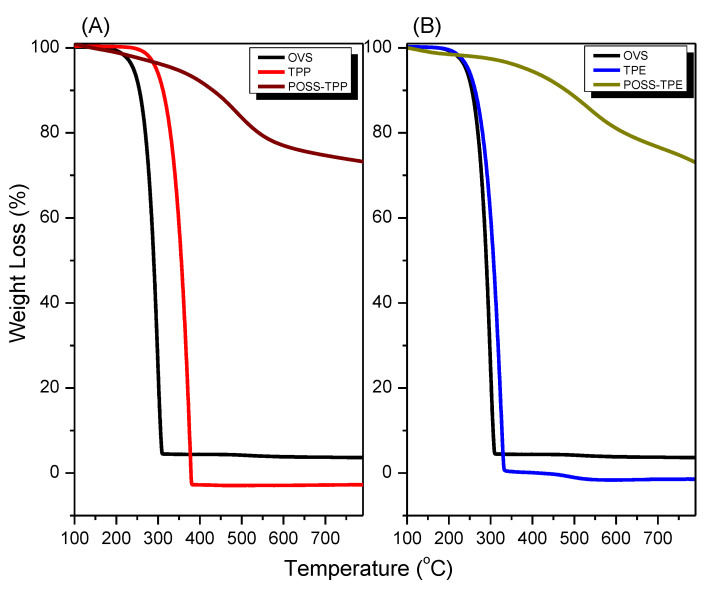
(**A**,**B**) TGA profiles of OVS, TPP, TPE, POSS-TPP, and POSS-TPE.

The thermal stabilities of OVS, TPP, TPE, POSS-TPP, and POSS-TPE were studied using TGA analysis under an N_2_ atmosphere, as shown in Figure 5. As displayed in Figure 5, the degradation temperatures (*T*_d5_ and *T*_d10_) and char yields were 242 °C, 255 °C, and 3.6% for OVS; 296 °C, 312 °C, and 0% for TPP; 246 °C, 263 °C, and 0% for TPE; 267 °C, 398 °C, and 71% for POSS-TPP; and 378 °C, 475 °C, and 73% for POSS-TPE, respectively. Interestingly, both POSS-TPP and POSS-TPE hybrid polymers displayed high thermal stabilities compared with their corresponding monomers, which was attributed to the introduction of POSS units, which enhanced the highly crosslinked network in the system, preventing the rapid degradation of hybrid polymers and also increasing the cleavage degradation of organic groups in the POSS-TPP and POSS-TPE. The values of *T*_d5_ and *T*_d10_ and the char yield for the OVS, TPP, TPE, and their corresponding polymers are summarized in Table 1.

**Figure 6:**
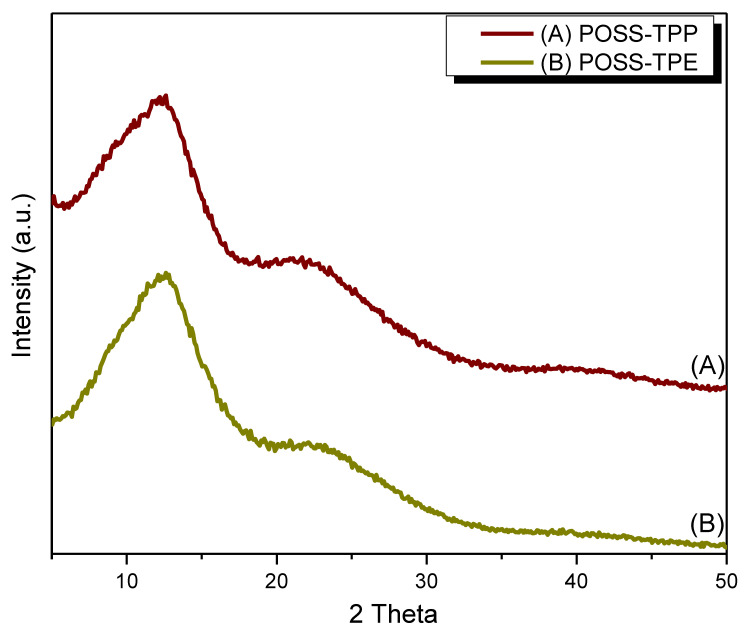
XRD pattern of (A) POSS-TPP and (B) POSS-TPE.

The wide-angle powder X-ray diffraction (WAXD) patterns of POSS-TPP and POSS-TPE (Figure 6) showed a broad peak at 2θ ≈ 22°, indicating that both of these two porous materials possessed amorphous characteristics, with no long-range order compared with the highly crystalline of TPE and OVS. These results displayed the random cross-linking between the TPP and TPE molecules with POSS cage structures [70,71].

**Figure 7:**
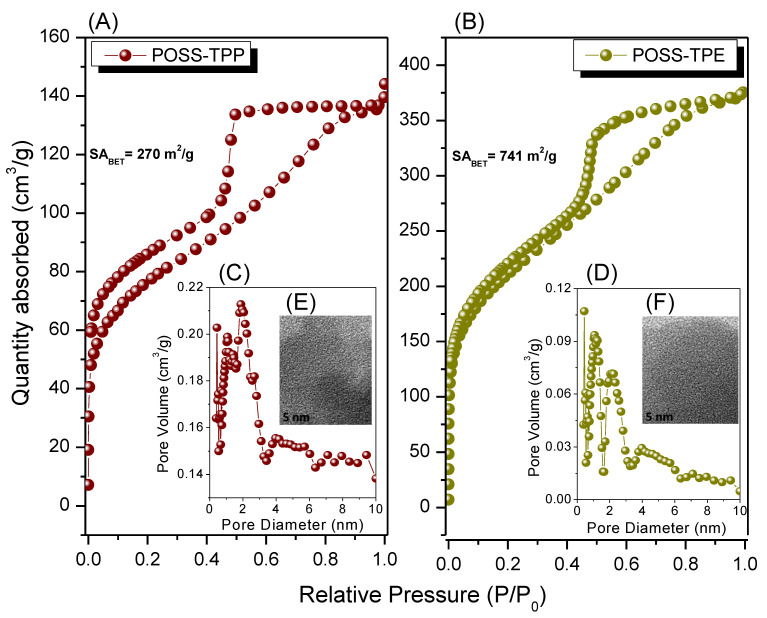
N_2_ adsoprtion and desorption measurements of (**A**) POSS-TPP and (**B**) POSS-TPE; pore size distributions of (**C**) POSS-TPP and (**D**) POSS-TPE; and TEM images of (**E**) POSS-TPP and (**F**) POSS-TPE.

Nitrogen adsorption–desorption experiments at 77 K were measured to examine the porosity parameters for our new POSS-TPP and POSS-TPE (Figure 7). Both of the nitrogen adsorption–desorption profiles of POSS-TPP and POSS-TPE (Figure 7A,B) revealed a rapid N_2_ uptake at a low P/P_0_ pressure that continued to increase at a high P/P_0_ pressure, indicating the coexistence of mesopores and micropores in the hybrid polymer network. Based on the International Union of Pure and Applied Chemistry (IUPAC) classification for BET curves, both POSS-TPP and POSS-TPE featured type IV adsorption isotherms. In addition, there were the hysteresis loops in both curves which also confirmed the presence of micropores and mesopores in the polymer framework. The Brunauer–Emmet–Teller (BET) surface area and total pore volume of the POSS-TPP and POSS-TPE were calculated to be 270 m^2^/g and 0.22 cm^3^/g, respectively, for POSS-TPP and 741 m^2^/g, 0.58 cm^3^/g, respectively, for POSS-TPE. The pore size distributions of POSS-TPP and POSS-TPE were calculated by using non-local density functional theory (NL-DFT), as elucidated in Figure 7C,D. The pore size diameters of the POSS-TPP were 1.04 nm and 1.82 nm (Figure 7C), while the POSS-TPE featured pore size diameters at 1.08 nm and 2.09 nm (Figure 7D). The porosity parameters of the POSS-TPP and POSS-TPE are provided in Table 1. In addition, TEM images (Figure 7E,F and Figure S5) of the POSS-TPP and POSS-TPE featured a microporous structure with no long-range order. The TEM results were consistent with the XRD pattern (Figure 6).

### 3.3. CO_2_ Capture and Iodine (I_2_) Adsorption Performance

**Figure 8:**
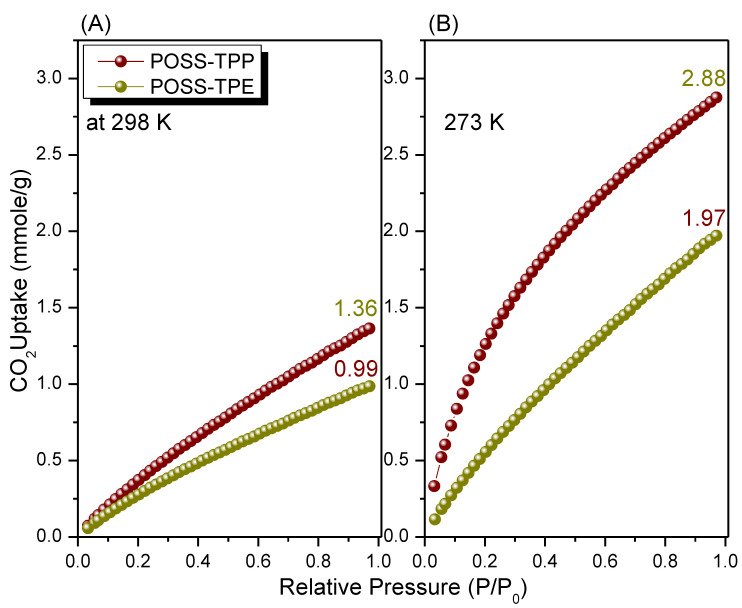
CO_2_ uptake profiles of (**A**) POSS-TPP and (**B**) POSS-TPE.

We applied POSS-TPP and POSS-TPE for CO_2_ capture and iodine adsorption due to their high total pore volumes, BET surface areas, and the presence of mesoporous and microporous structures in the polymer networks. We performed CO_2_ uptake measurements for POSS-TPP and POSS-TPE at 298 K and 273 K to examine their CO_2_ uptake abilities, as presented in Figure 8. The results revealed that POSS-TPP displayed the highest CO_2_ adsorption capacity, reaching 1.63 mmol/g at 298 K and 2.88 mmol/g at 273 K, while POSS-TPE showed CO_2_ capture values reaching 0.99 mmol/g at 298 K and 1.97 mmol/g at 273 K. As previously reported, the enhancement of polymeric porous materials for the adsoprtion of acidic CO_2_ gas can be achieved through the incorporation of basic nitrogen functionalities and oxygen groups into the polymeric network. Interestingly, even POSS-TPP possesses a low BET surface area (270 m^2^/g) compared with the POSS-TPE. POSS-TPP displayed a higher CO_2_ uptake than that other porous materials, such as poly(1,1-dimethyl-3,4-diphenyl-2,5-bis(4’,4’-diphenylaminophenyl)) silole (PDMTPAS), (1.02 mmol/g at 298 K), An-CPOP-1 (1.30 mmol at 298 K and 1.40 mmol/g at 273 K), An-CPOP-2 (1.40 mmol/g at 298 K and 1.52 mmol/g at 273 K), and C2M1-Al (1.44 mmol/g at 273 K) [5,72,73], due to the POSS-TPP containing N-atoms, which can lead to effectively increasing the dipole–quadrupole interactions between the CO_2_ molecules and the POSS-TPP. The CO_2_ adsoprtion capacity performance of POSS-TPP and POSS-TPE, compared with other porous materials, is provided in Table S1.

**Figure 9:**
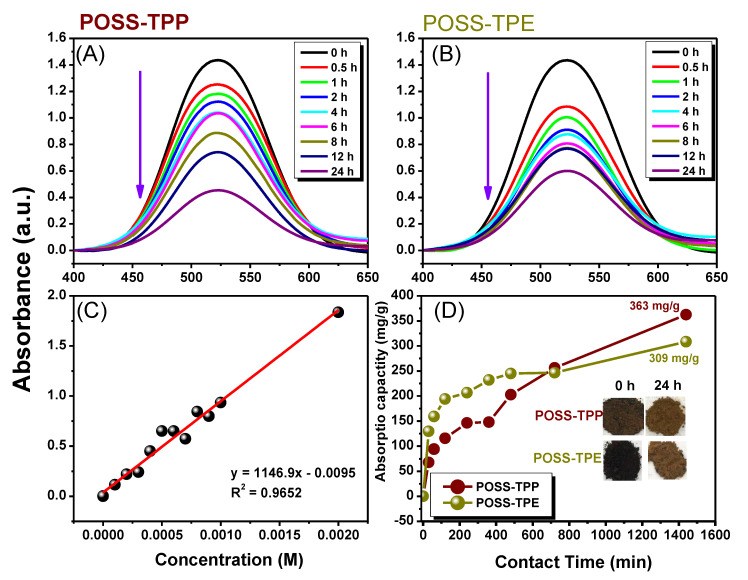
(**A**,**B**) UV-Vis absorption profile of the iodine solution after stirring POSS-TPP and POSS-TPE for different times (0–24 h). (**C**) The calibration curve of iodine was measured through recording the UV-Vis spectra of different I_2_ concentrations in hexane. (**D**) Maximum absorption capacity of the iodine by the absorbents of POSS-TPP and POSS-TPE.

The developing porous polymer absorbents featuring good porosities and unique structures to decrease environmental pollution, such as I_2_, have attracted much intention in both academics and industry [19]. As reported by different groups, the performance of the porous organic polymers as adsorbents for iodine uptake strongly depends on the interaction of the iodine molecule with the polymer framework and does not depend on the pore size or volume of the polymer network [19]. Figure 9A,B presents the UV absorbance spectra of the suspension solutions of the POSS-TPP and POSS-TPE in ethanol with an I_2_ solution (10^−4^ M) at different interval times (0 h to 24 h). As shown in Figure 9A,B, the maximum intensity of the adsorption peak of I_2_ was detected at λ_max_ = 523 nm. After the addition of 5 mg of POSS-TPP or POSS-TPE into the iodine solution in hexane, the intensity of the absorption bands of the I_2_ gradually decreased when the time increased from 0 to 24 h. These results suggest that both POSS-TPP and POSS-TPE could act as good precursors for I_2_ adsoprtion. The calibration curve (Figure 9C) of the iodine was measured through the recording of UV-Vis spectra of different I_2_ concentrations in hexane. We observed that the maximum adsorption capability value (Figure 9D) of the POSS-TPP (363 mg/g) was higher than that of POSS-TPP (309 mg/g). From the adsoprtion experiments, the POSS-TPP was considered a better adsorbent material for iodine than POSS-TPE due to the presence of N-atoms in the POSS-TPP framework, leading to strong interaction with the I_2_ molecules. POSS-TPP and POSS-TPE showed excellent iodine uptake performance compared with other POP adsorbents, as presented in Table S2. In addition, the synthesis of hybrid porous organic polymers based on the POSS unit through a simple Friedel–Crafts reaction with large-scale applications was considered as an effective synthetic method [36]. For recycling experiments, the iodine-loaded POSS-TPP and POSS-TPE samples were washed with ethanol to remove the I_2_ molecules and dried at 100 °C. As shown in Figure S6, after recycling the measurements six times, the iodine capture capacities of the POSS-TPP and POSS-TPE were found to drop from 363 mg/g to 362 mg/g and 309 mg/g to 308 mg/g, respectively. From these results, our new adsorbent materials were used as good precursors for reversible and recyclable iodine uptake.

## 4. Conclusions

In summary, two different kinds of hybrid porous materials (POSS-TPP and POSS-TPE) were easily synthesized via simple Friedel−Crafts polymerization of cubic octavinylsilsesquioxane as node building blocks with tetraphenylpyrazine and tetraphenylethene, respectively, in the presence of FeCl_3_ as a catalyst. The synthesized POSS-TPP and POSS-TPE hybrid porous polymers exhibited high surface areas of 270 m^2^/g and 741 m^2^/g, respectively, with mesoporous structures. In addition, the POSS-TPP displayed the highest CO_2_ adsorption capacity, reaching 1.63 mmol/g at 298 K and 2.88 mmol/g at 273 K, with a maximum adsorption capacity for I_2_ reaching 363 mg/g, compared with the POSS-TPE and other porous materials.

## Figures and Tables

**Table 1:** Thermal stability and porosity properties of OVS, TPP, TPE, and their corresponding polymers.

Sample	*T*_d5_ (°C)	*T*_d10_ (°C)	Char Yield (wt.%)	Surface Area (m^2^/g)	Pore Size (nm)
OVS	242	255	4	–	–
TPP	296	312	0	–	–
TPE	246	263	0	–	–
POSS-TPP	267	398	71	270	1.04, 1.86
POSS-TPE	378	475	73	741	1.08, 2.09

## Data Availability

The data presented in this study are available on request from the corresponding author.

## Supplementary Materials

The following are available online at https://www.mdpi.com/2073-4360/13/2/221/s1. Figure S1: ^1^H NMR spectrum of TPP. Figure S2: ^13^C NMR spectrum of TPP. Figure S3: ^1^H NMR spectrum of TPE. Figure S4: ^13^C NMR spectrum of TPE. Figure S5: TEM images of POSS-TPP (A,B,C) and POSS-TPE (D,E,F). Figure S6: Repeated I_2_ uptake experiments for (A) POSS-TPP and (B) POSS-TPE. Table S1: Performance data of POSS-TPP and POSS-TPE compared with those of other previously porous materials. Table S2: Iodine uptake properties of POSS-TPP, POSS-TPE and other porous materials.
