# Supplementary material for: Multifunctional Polyhedral Oligomeric Silsesquioxane (POSS) Based Hybrid Porous Materials for CO2 Uptake and Iodine Adsorption
Source: Polymers (Basel). 2021 Jan 10;13(2):221. doi: 10.3390/polym13020221 (PMC7826546; doi:10.3390/polym13020221)
Supplement: Supplementary file 1 [file polymers-13-00221-s001.pdf]

Supporting Information for

Multifunctional Polyhedral Oligomeric Silsesquioxane (POSS) Based Hybrid Porous Materials for CO_2 Uptake and Iodine Adsoprtion

Mohamed Gamal Mohamed[1,2], Mei-Yin Tsai[1], Chih-Feng Wang[3], Chih-Feng Huang[4], Martin Danko,[5] Lizong Dai[6], Tao Chen[7] and Shiao-Wei Kuo[1,8,*]

[1] Department of Materials and Optoelectronic Science, Center of Crystal Research, National Sun Yat-Sen University, Kaohsiung 80424, Taiwan.; mgamal.eldin34@gmail.com and m073100011@student.nsysu.edu.tw
[2]Chemistry Department, Faculty of Science, Assiut University, Assiut 71516, Egypt.
[3]Advanced Membrane Materials Research Center, Graduate Institute of Applied Science and Technology, National Taiwan University of Science and Technology, Taipei 10607, Taiwan; cfwang@mail.ntust.edu.tw
[4]Department of Chemical Engineering, i-Center for Advanced Science and Technology (iCAST), National Chung Hsing University, Taichung 40227, Taiwan; huangcf@dragon.nchu.edu.tw
[5]Department of Synthesis and Characterization of Polymers, Polymer Institute, Slovak Academy of Sciences, Dúbravská cesta 9, Bratislava 84541, Slovakia. Martin.Danko@savba.sk
[6]Fujian Provincial Key Laboratory of Fire Retardant Materials, College of Materials, Xiamen University, Xiamen 361005, China; lzdai@xmu.edu.cn
[7]Ningbo Institute of Material Technology and Engineering, Chinese Academy of Science, Zhongguan West Road 1219, 315201 Ningbo, China; tao.chen@nimte.ac.cn
[8]Department of Medicinal and Applied Chemistry, Kaohsiung Medical University, Kaohsiung 807, Taiwan.

* Correspondence: kuosw@faculty.nsysu.edu.tw; Tel.: +886-7-525-4099

Characterization

FTIR spectra were recorded using a Bruker Tensor 27 FTIR spectrophotometer and the conventional KBr disk method; 32 scans were collected at a spectral resolution of 4 cm^{-1}. The films used in this study were sufficiently thin to obey the Beer-Lambert law. Wide-Angle X-ray diffraction (WAXD) pattern was obscured from the wiggler beamline BL17A1 of the National Synchrotron Radiation Research Center (NSRRC), Taiwan. A triangular bent Si (111) single crystal was used to obtain a monochromated beam having a wavelength (λ) of 1.33 Å. Cross-polarization with MAS (CP/MAS) was used to acquire ^{13}C NMR spectral data at 75.5 MHz. The CP contact time was 2 ms; $_{1}H$ decoupling was applied during data acquisition. The decoupling frequency corresponded to 32 kHz. The MAS sample spinning rate was 10 kHz. Transmission electron microscope (TEM) images were obtained with a JEOL JEM-2010 instrument operated at 200 kV. Field emission scanning electron microscopy (FE-SEM) was conducted using a JEOL JSM7610F scanning electron microscope. Samples were treated via Pt sputtering for 100 s before observation. BET surface area and porosimetry measurements of the prepared samples (ca. 40-100 mg) were performed using a BEL. Nitrogen isotherms were generated through incremental exposure to ultrahigh-purity N_2 (up to ca. 1 atm) in a liquid nitrogen (77 K) bath. Surface parameters were determined using BET adsorption models in the instrument's software. TGA was performed using a TA Q-50 analyzer under a flow of N_2 atmosphere. The samples were sealed in a Pt cell and heated from 40 to 800 °C at a heating rate of 20 °C min^{-1} under a flow of N_2 atmosphere at a flow rate of 60 mL min^{-1}.UV–Vis spectra were recorded at 25 °C using a Jasco V-570 spectrometer, with deionized water as the solvent.

Table S1: Performance data of POSS-TPP and POSS-TPE compared with those of other previously porous materials.

**Samples**	**CO_2 uptake (mmole/g)**		**Ref**
	298 K	273 K	
**PDMTPAS**	1.02	1.76	[1]
**PDPTPAS**	1.04	1.76	[1]
**An-HPP**	0.52	1.29	[2]
**TPT-HPP**	0.90	1.99	[2]
**Car-HPP**	1.24	2.29	[2]
**TPE-HPP**	0.85	1.49	[2]
**HPP-1c**	0.86	1.56	[3]
**LHPP-3**	0.77	1.44	[4]
**HPP-3**	-	1.42	[5]
**THPP**	-	1.16	[6]
**PHAP-1**		2.60	[7]
**PECONF-4**		0.14	[8]
**POSS-TPP**	1.63	2.88	This work
**POSS-TPE**	0.99	1.97	This work

Table S2. Iodine uptake properties of POSS-TPP, POSS-TPE and other porous materials.

Sample	Surface area ($m^{2/}g$)	Iodine uptake (mg/g)	Ref
Activated carbon	-	300	[9]
CC3	-	364	[10]
NOP-54	1187	202	[11]
ZIF-8	1875	1200	[12]
Ag@Mon-MOF	690	250	[13]
Ag@Zeolite Mordenities	-	275	[14]
HCMP-3	82	3160	[15]
PAF-1	2081	1860	[16]
TTPT	315.5	1770	[17]
pha-HCOPs	217.31	1310	[18]
POSS-TPP	270	363	This work
POSS-TPE	741	309	This work

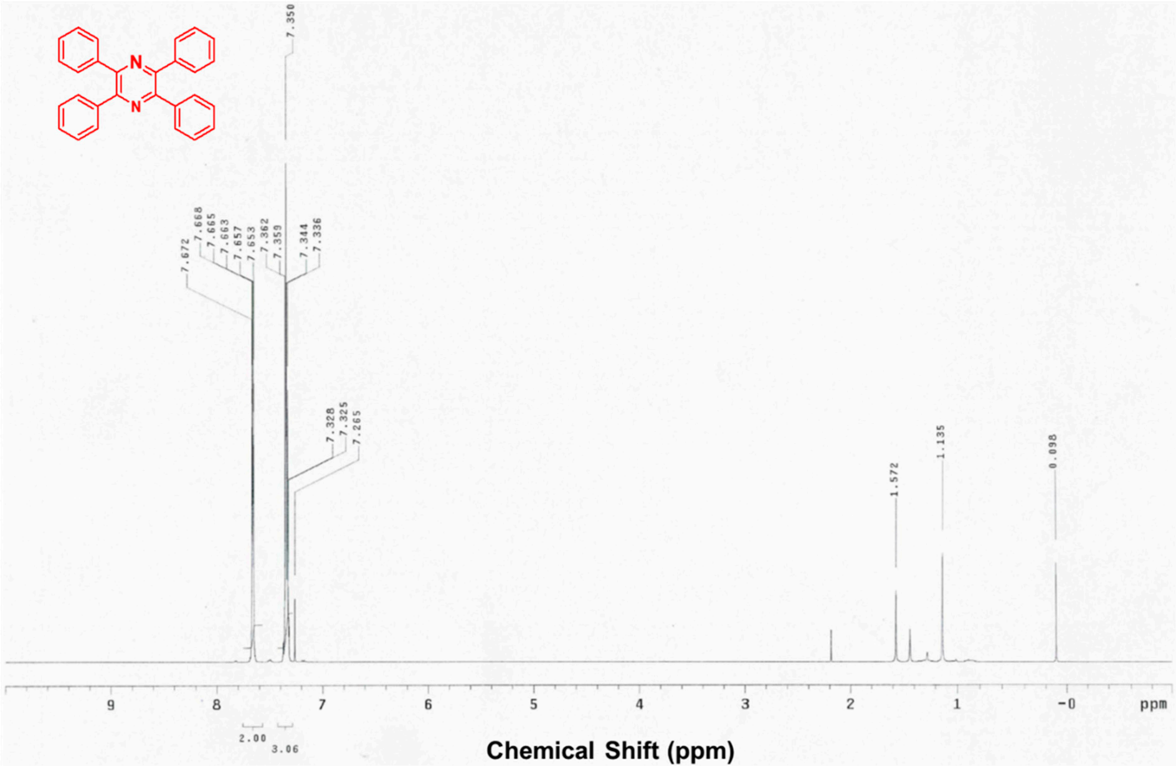

Figure S1. ^{1}H NMR spectrum of TPP.

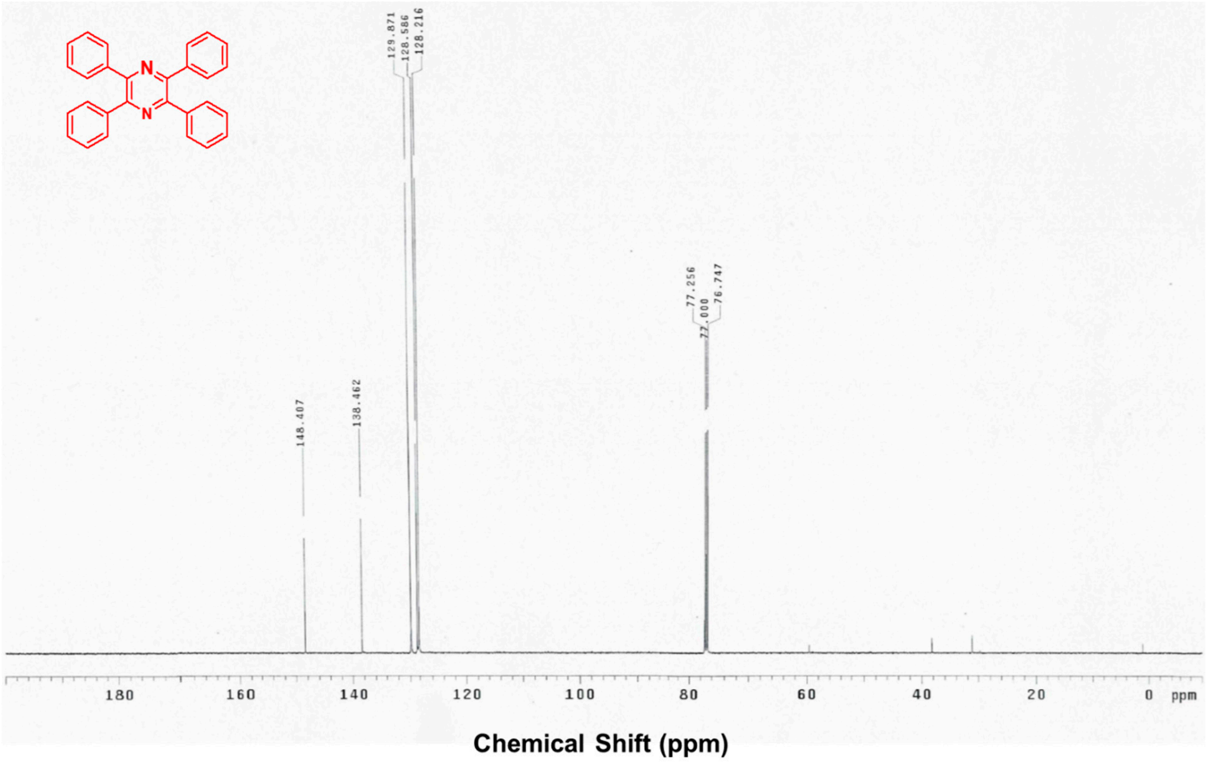

Figure S2. ^{13}C NMR spectrum of TPP.

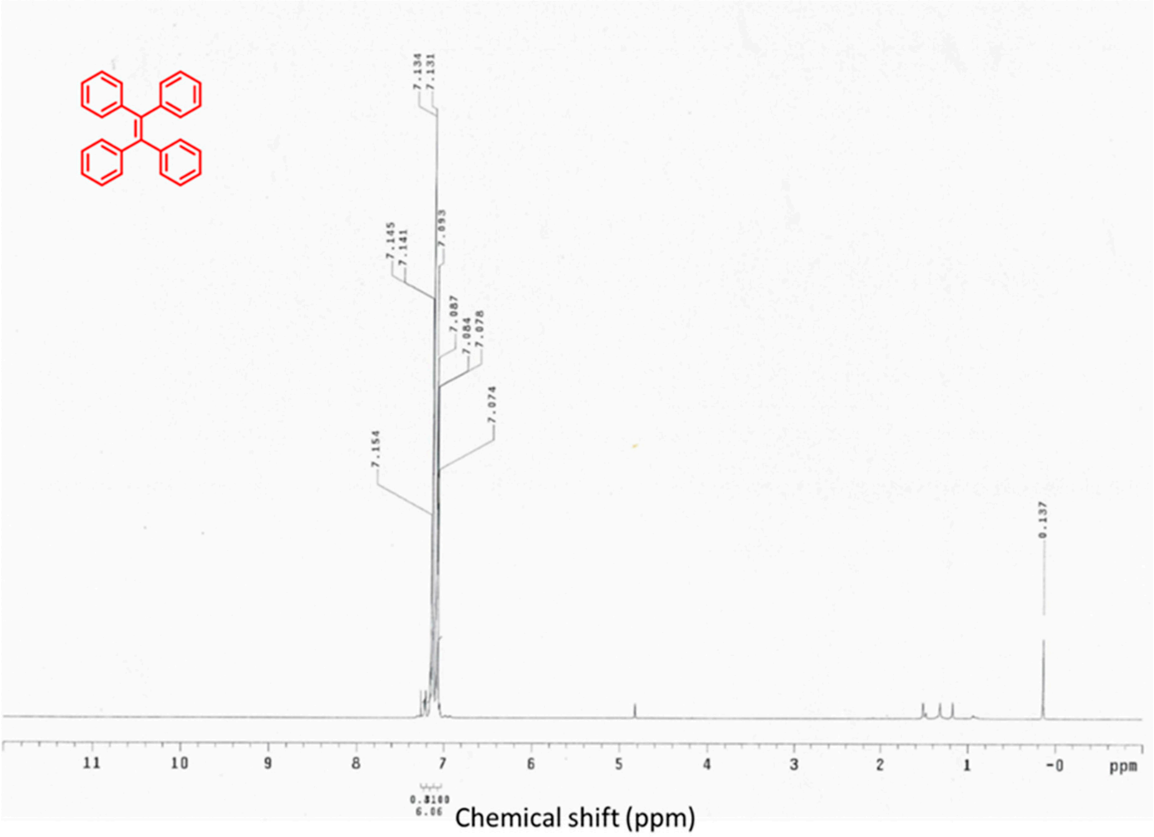

Figure S3. ^{1}H NMR spectrum of TPE.

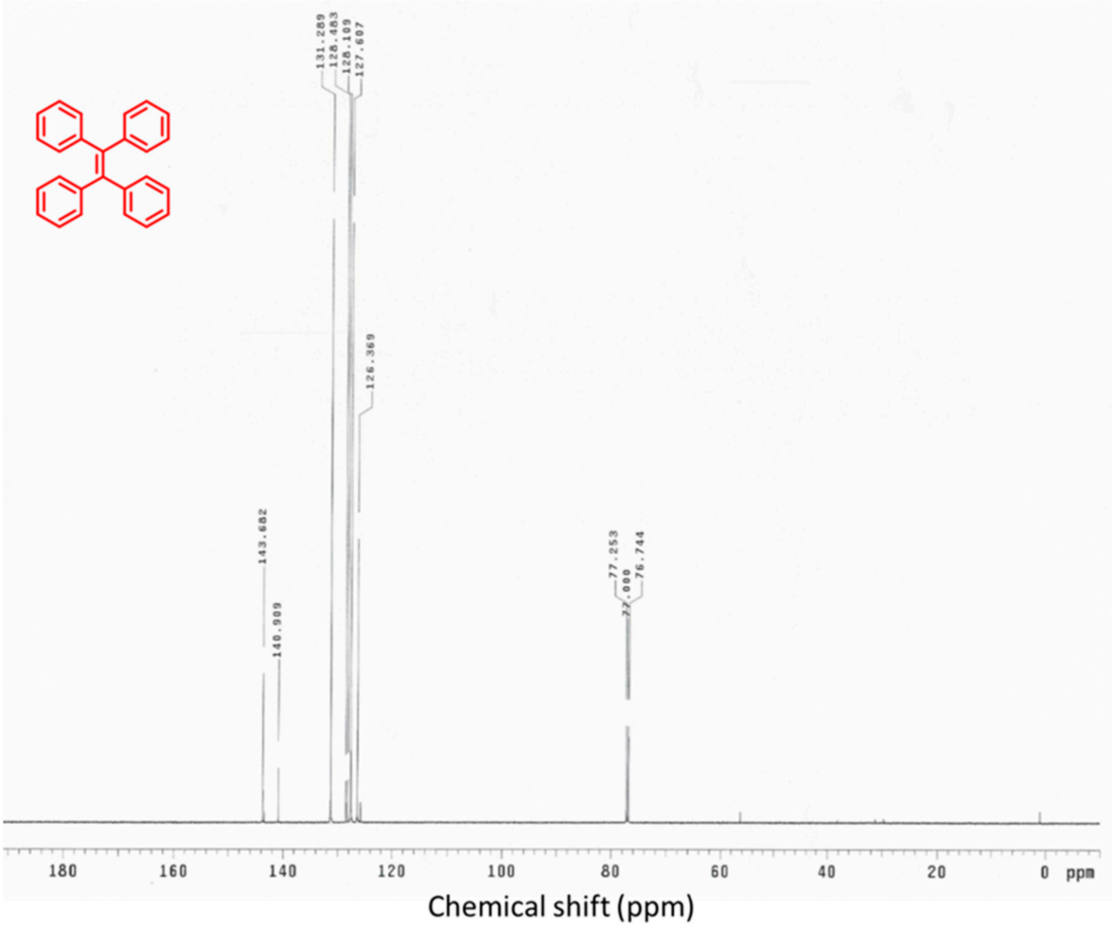

Figure S4. ^{13}C NMR spectrum of TPE.

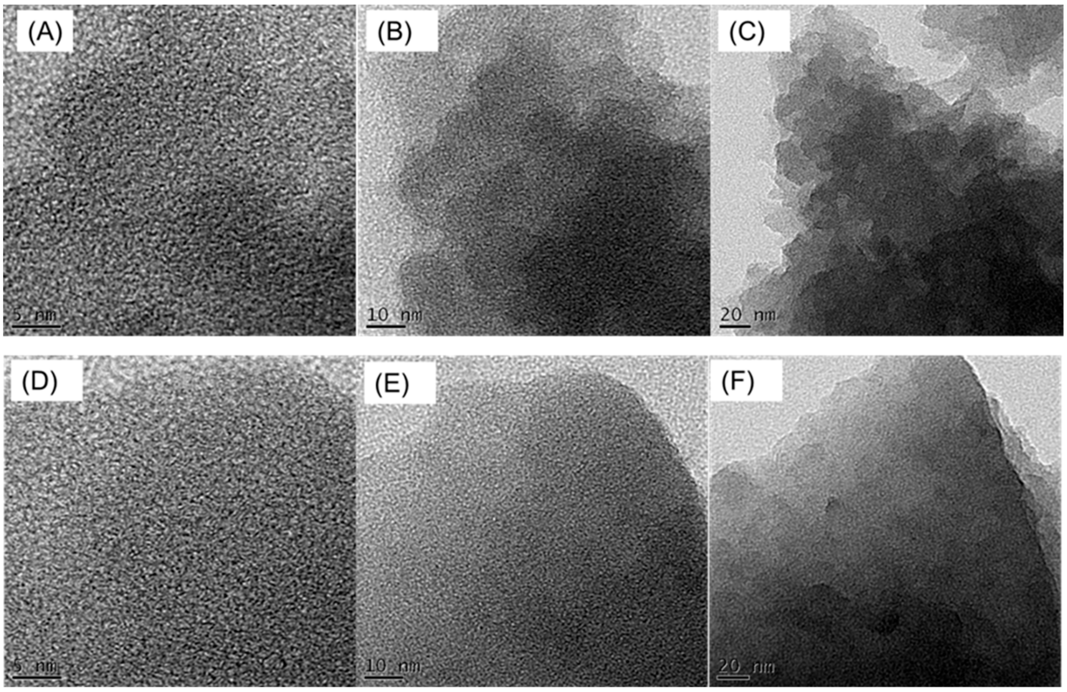

Figure S5. TEM images of POSS-TPP (A, B, C) and POSS-TPE (D, E, F).

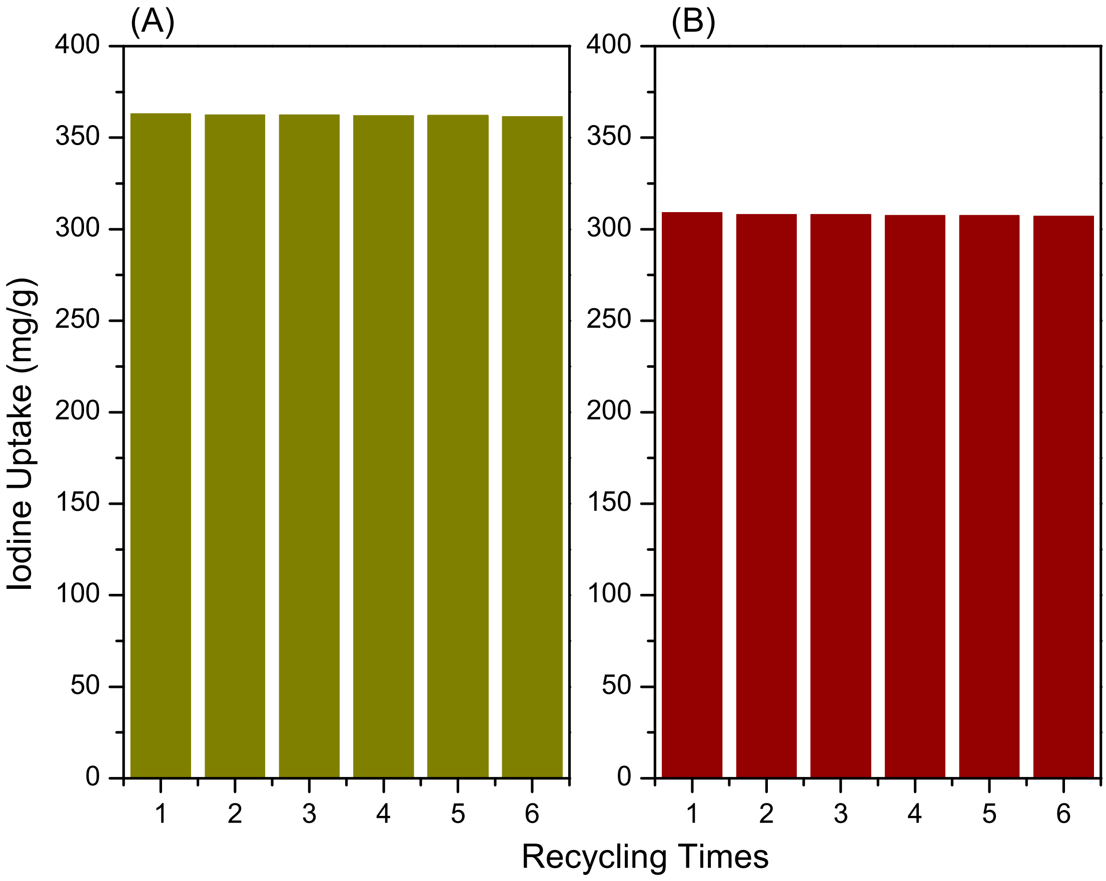

Figure S6. Repeated I_2 uptake experiments for (A) POSS-TPP and (B) POSS-TPE.

References

[1] Shu, G.; Zhang, C.; Li, Y.; Jiang, J.X.; Wang, X.; Li, H.; Wang, F. Hypercrosslinked silole-containing microporous organic polymers with N-functionalized pore surfaces for gas storage and separation. *J. Appl. Polym. Sci.* **2018**, *135*, 45907.

[2] Mohamed, M.G.; Liu, N.Y.; EL-Mahdy, A.F.M.; Kuo. S.W. Ultrastable luminescent hybrid microporous polymers based on polyhedral oligomeric silsesquioxane for CO_2 uptake and metal ion sensing. *Micropor. Mesopor. Mater*. **2021**, *311*, 110695.

[3] Wang, D.; Feng, S.; Liu, H. Fluorescence-Tuned Polyhedral Oligomeric Silsesquioxane-Based Porous Polymers. *Chem. Eur. J.* **2016**, *22*, 14319-14327.

[4] Wang, D.; Li, L.; Yang, W.; Zuo, Y.; Feng, S.; Liu, H. POSS-based luminescent porous polymers for carbon dioxide sorption and nitroaromatic explosives detection. *RSC Adv*., **2014**, *4*, 59877-59884.

[5] Liu, H.; Liu, H. Selective dye adsorption and metal ion detection using multifunctional silsesquioxane-based tetraphenylethene-linked nanoporous polymers. *J. Mater. Chem. A*. **2017**, *5*, 9156-9162.

[6] Liu, H.; Ge, M. A silsesquioxane-based thiophene-bridged hybrid nanoporous network as a highly efficient adsorbent for wastewater treatment. *J. Mater. Chem. A*. **2016**, *4*, 16714-16722.

[7] Puthiaraj, P.; Ahn, W.S. CO_2 Capture by Porous Hyper-Cross-Linked Aromatic Polymers Synthesized Using Tetrahedral Precursors. *Ind. Eng. Chem. Res*. **2016**, *65*, 7917-7923.

[8] Mohanty, P.; Kull, L.D.; Landskron, K. Porous Covalent Electron-Rich Organonitridic Frameworks as Highly Selective Sorbents for Methane and Carbon Dioxide. *Nat. Commun*. **2011**, *2*, 401-406.

[9] Ma, H.; Chen, J. J.; Tan, L.; Bu, J. H.; Zhu, Y.; Tan, B.; Zhang, C. Nitrogen-Rich Triptycene-Based Porous Polymer for Gas Storage and Iodine Enrichment. *ACS Macro Lett.* **2016**, *5*, 1039–1043.

[10] Hasell, T.; Schmidtmann, M.; Cooper, A. I. Molecular Doping of Porous Organic Cages. *J. Am. Chem. Soc.* **2011**, *133*, 14920–14923.

[11] Chen, D.; Fu, Y.; Yu, W.; Yu, G.; Pan, C. Versatile Adamantane-Based Porous Polymers with Enhanced Microporosity for Efficient CO_2 Capture and Iodine Removal. *Chem. Eng. J.* **2018**, *334*, 900–906.

[12] Sava, D. F.; Garino, T. J.; Nenoff, T. M. Iodine Confinement into Metal–Organic Frameworks (MOFs): Low-Temperature Sintering Glasses To Form Novel Glass Composite Material (GCM) Alternative Waste Forms. *Ind. Eng. Chem. Res.* **2012**, *51*, 614–620.

[13] Katsoulidis, A. P.; He, J.; Kanatzidis, M. G. Functional Monolithic Polymeric Organic Framework Aerogel as Reducing and Hosting Media for Ag Nanoparticles and Application in Capturing of Iodine Vapors. *Chem. Mater.* **2012**, *24*, 1937–1943.

[14] Chapman, K. W.; Chupas, P. J.; Nenoff, T. M. Radioactive Iodine Capture in Silver-Containing Mordenites through Nanoscale Silver Iodide Formation. *J. Am. Chem. Soc.* **2010**, *132*, 8897–8899.

[15] Liao, Y.; Weber, J.; Mills, B. M.; Ren, Z.; Faul, C. F. J. Highly efficient and reversible iodine capture in hexaphenylbenzene-based conjugated microporous polymers. *Macromolecules*, **2016**, *49*, 6322–6333.

[16] Pei, C.; Ben, T.; Xu, S.; Qiu, S. Ultrahigh iodine adsorption in porous organic frameworks. *J. Mater. Chem. A*, **2014**, *2*, 7179–7187.

[17] Geng, T.; Zhu, Z.; Zhang, W.; Wang, Y. A nitrogen-rich fluorescent conjugated microporous polymer with triazine and triphenylamine units for high iodine capture and nitro aromatic compound detection. J. Mater. Chem. A, **2017**, *5*, 7612–7617.

[18] Lin, L.; Guan, H.; Zou, D.; Dong, Z.; Liu, Z.; Xu, F.; Xie, Z.; Li, Y. A pharmaceutical hydrogen-bonded covalent organic polymer for enrichment of volatile iodine. *RSC Adv.*, **2017**, *7*, 54407–54415.
